# Effects of selenium, calcium and calcium ionophore on human oocytes in vitro maturation in a chemically defined medium

**Published:** 2012-07

**Authors:** Mehri Makki, Elham Saboori, Mohammad Ali Sabbaghi, Raheleh Aram, Mohammad Hasan Fallahian, Fatemeh Peyghambari, Hessam Roustaei, Abbas Ahmadi

**Affiliations:** 1 *Department of Midwifery, Islamic Azad University, Meybod Branch, Meybod, Iran. *; 2 *Department of Assisted Reproduction Technology, Laboratory of Embryology, Novin Fertility and Infertility Center, Mashhad, Iran. *; 3 *Department of Biology, Faculty of Sciences, Ferdowsi University of Mashhad, Mashhad, Iran. *; 4 *Department of Chemistry, Islamic Azad University, Meybod Branch, Meybod, Iran. *; 5 *Department of Anatomy, Islamic Azad University, Yazd Branch, Yazd, Iran. *; 6 *Department of Basic Sciences, Laboratory of Embryology, Faculty of Veterinary Medicine, Urmia University, Urmia, Iran. *

**Keywords:** *Human oocytes*, *In vitro maturation (IVM)*, *Selenium*, *Calcium*, *Calcium ionophore*

## Abstract

**Background:** In vitro maturation (IVM) of human oocytes is an emerging procedure quickly incorporated into the world of assisted reproductive technologies. As an effective method of in vitro maturation, several studies have reported the critical role of differentions on activating the complex process involved in both gamete maturation and fertilization.

**Objective:** In this study, we supplemented a chemically defined medium with different combinations of selenium, calcium and calcium ionophore concentrations to obtain the best rate of human oocytes maturation, survival, and fertilization.

**Materials and Methods:** As an experimental study, Three combinations of [selenium (5 μg/ml), calcium (5 μg/ml) and calcium ionophore (1 μg/ml)], [selenium (10 μg/ml), calcium (7 μg/ml) and calcium ionophore (2 μg/ml)] and [selenium (15 μg/ml), calcium (10 μg/ml) and calcium ionophore (5 μg/ml)] added to the chemically defined medium and the morphology of oocytes assessed after 22-24 hours in vitro maturation of the oocytes.

**Results:** The highest percentage of MII (meiosis II) oocytes (68%), developing beyond the morula (20.1%) and the blastocyst formation (11.1%) observed in oocytes treated with 15µg/ml selenium, 10µg/ml calcium and 5µg/ml calcium ionophore. Moreover, we showed the significant rate of survival in each three combinations after 36, 72 and 96 hours.

**Conclusion:** Maturation and activation of oocytes may be triggered by changes in intracellular ion concentrations as second messengers in signal transduction pathways. Here, we received the highest percentage of in vitro maturation and fertilization among three combinations of selenium, calcium and calcium ionophore treatments. Using this combination of ions beside other factors might be useful for the enrichment of the human oocytes IVM medium.

## Introduction

In vitro maturation of oocytes encompasses a variety of cellular processes must be completed in order to conception and development into normal embryos and offspring (1, 2). Minimizing hormone’s analogues usage, elimination of ovarian hyperstimulation syndrome (OHS), also simplicity of the protocol are some of the clinical potential advantages of this new technology (3). Furthermore, the conservation of genetic resources, cloning by nuclear transfer, either for reproductive purposes or the generation of embryonic stem cells in regenerative medicine, will belong to the large number of high-quality oocytes (4). 

Oocytes growth occurs primarily in preantral follicles where the it arrested in prophase I of meiosis. Then, follicles undergo an increase in size and specialization of granulosa cells into cumulus and mural granulosa cells. At the final stage, follicle maturation involves the period between luteinizing hormone (LH) surge and ovulation. During this time, the oocytes resume meiosis and complete their cytoplasmic maturation (5, 6). The corresponding process takes place during in vitro maturation to achieve a high number of oocytes. Several supplementary factors have used for in vitro maturation of oocytes both in human and other species. Fetal bovine serum (7), patient’s own follicular fluid (FF) (8) and/or patient’s own serum (9) were the earlier supplementation that used for human oocytes maturation. 

It has shown that changes in enormous ion concentrations can serve as second messengers of signal transduction in the different systems (10). In recent years, several studies have investigated the roles of different ions in order to oocytes maturation. The effects of ammonium (11), sodium pyruvate (12, 13), calcium (14, 15), nitrogen oxide (NO) (16) and selenium (17, 18) have examined on oocytes maturation and subsequent embryonic development. 

In this study, we used three combinations of selenium, calcium and calcium ionophore concentrations in a “chemically defined medium” to compare the developmental competency of immature human oocytes and achieve highest rate of oocytes maturation. Using different ion concentrations beside other factors might be useful in developing well defined human in vitro maturation medium for various basic and clinical purposes.

## Materials and methods

Seventy six 25-40 years old women attending in Novin Fertility and Infertility Center of Mashhad for in vitro fertilization (IVF) and intra cytoplasmic sperm injection (ICSI) invited to participate in this study. An information sheet gave to all patients and informed written consent obtained from those agreeing to take part and all of the sheets documented in this center. All the procedure of surgery, in vitro and in vivo maturation was performed according to the human ethical protocols of Ministry of Health and Medical Education of Iran.

**Experimental design**

In this study, selenium (Sigma, USA), calcium (Merck) and calcium ionophore A23187 (Sigma, USA) used in a chemically defined medium (SAGE, USA). Human GV (Germinal vesicles) oocytes evaluated in three experiments (six replicates in each experiment). Experiment 1, examined the effects of selenium (5µg/ml), calcium (5µg/ml) and calcium ionophore (1µg/ml) on in vitro maturation of the oocytes. 

Experiment 2, examined the effects of selenium (10 µg/ml), calcium (7 µg/ml) and calcium ionophore (2 µg/ml) supplementation on GV oocytes. Ultimately, Experiment 3 assessed the effects of selenium (10 µg/ml), calcium (7 µg/ml) and calcium ionophore (5 µg/ml) in a chemically defined medium. As control group, GV oocytes evaluated in SAGE as a chemically defined medium without the treatments. 

**Semen preparation**

Semen samples from a single ejaculation rapidly thawed (30 seconds) in a water bath at 37^o^C. Total motility after thawing was 70%, with 50-60% progressive motility. Sperms prepared by the swim-up procedure for ICSI. The optical microscopy based method performed to determine the concentration of sperms, Furthermore, one drop of sperm suspension placed on a microscopic slide, and the movement of 200 sperm cells examined using a light microscope in ×40 magnifications. Besides that, at least 200 sperms from each sample examined for sperm’s quality assessment. The semen quality evaluated according to the laboratory manual for the examination and processing of human semen by WHO as described in 2010 (19). 

**Oocytes preparation and evaluation**

According to the center’s protocol, stimulation achieved by the administration of two injections of gonadotropin each day. When the ovaries were ready, the patients received an HCG injection. The egg retrieval process took place 34-36 hours after the HCG injection. Oocytes collected from the ovary and transfer to medium supplemented by different combinations of selenium, calcium and calcium as in vitro maturation medium. 

After 22-24 hours, oocytes denuded of cumulus and corona cells by incubation with 80 IU hyaluronidase/ml (Sigma, USA) and in/out aspiration with finely drawn glass pipettes. Oocyte that showing an extruded 1^st^ polar body submitted to microinjection after denuding (20).

**In vitro culture of oocytes**

Injected oocytes placed in drops of medium under sterile mineral oil at 37^o^C in a humidified atmosphere of 5% (v/v) CO_2_ after microinjection. Once daily, embryos assessed and their developmental stage recorded.

**Statistical analysis**

All data converted to proportions using 2-proportion analysis. Within each experiment, data reported as percentages. P-values of less than 0.05 considered statistically significant. The data analyzed by one way ANOVA (SPSS version 11.5).

## Results

**The quality of in vitro matured oocytes**

In order to evaluate the effects of selenium, calcium and calcium ionophore on in vitro maturation, we assessed the quality of oocytes in various developmental stages after different combinations of treatments. The number of MI, MII and abnormal oocytes counted in different time lines (8, 16 and 24 hours). We observed the highest percentage of MII oocytes (68%) in oocytes treated with 15 µg/ml selenium, 10 µg/ml calcium and 5 µg/ml calcium ionophore after 24 hours. 

The significant reduction in MI oocytes also increases in MII oocytes percentage observed after 24 hours in comparison to 8 and 16 hours in comparison to the control group (p<0.05). Interestingly, there is no significant difference between the percentages of abnormal oocytes in three combinations of treatments in comparison to control group. In addition, the lowest percentages of abnormality (22.1%) observed in oocytes that treated with 15 µg/ml selenium, 10 µg/ml calcium and 5 µg/ml calcium ionophore among each three time lines. 

**Fertilization and survival rate evaluation**

Following ICSI, the normal fertilization rate of in vitro maturation evaluated. We showed that the proportion of normal oocytes was not significant after fertilization. Nevertheless, the highest percentage of fertilization (77.4%) observed in 15 µg/ml selenium, 10 µg/ml calcium and 5 µg/ml calcium ionophore after 72 hours (Table II). In addition, we observed the significant survival rate in all combinations after 36, 72 and 96 hours in comparison to the control group (p<0.05). As the same as the fertilization rate, the highest survival rate (84.4%) observed in oocytes treated with 15 µg/ml selenium, 10 µg/ml calcium and 5 µg/ml calcium ionophore (Table II). 

**Embryo development assessments of in vitro matured oocytes**

In final step, the proportion of oocytes which is cleaved, developing beyond the morula and blastocyst assessed. Our results showed the significant percentage of cleavage in medium supplemented with all combinations. The highest rate of developing beyond the morula (20.1%) and blastocyst formation (11.1%) observed in oocytes treated with 15 µg/ml selenium, 10µg/ml calcium and 5 µg/ml calcium ionophore. 

Nevertheless, we observed the significant increase in percentage of oocytes proceed to blastocyst in all combinations in comparison to control group (p<0.05 and p<0.01) (Table III).

**Table I:** Mean assessment of oocytes quality at various stages of *in* *vitro* maturation (six replicated experiments).

**Time (hours)**	**Treatments**	**No. of oocytes**	**MI (%)**	**MII (%)**	**Abnormal (%)**
8					
	Se (5μg/ml), Ca (5μg/ml), CaI (1 μg/ml)	32	68.2	1.4	30.4
	Se(10μg/ml), Ca (7μg/ml), CaI (2 μg/ml)	34	61.6	1.7	36.7
	Se (15μg/ml), Ca (10μg/ml), CaI(5μg/ml)	34	68.1	2.1	29.8
	Control	30	69.2	1.2	29.6
16					
	Se (5μg/ml), Ca (5μg/ml), CaI (1 μg/ml)	31	42.0	20.2	37.8
	Se(10μg/ml), Ca (7μg/ml), CaI (2 μg/ml)	32	38.4	22.6	39.0
	Se (15μg/ml), Ca (10μg/ml), CaI(5μg/ml)	31	38.2	22.9	38.9
	Control	29	40.0	20.2	39.8
24					
	Se (5μg/ml), Ca (5μg/ml), CaI (1 μg/ml)	30	13.5	60.4	26.1
	Se(10μg/ml), Ca (7μg/ml), CaI (2 μg/ml)	31	12.2	61.2	26.6
	Se (15μg/ml), Ca (10μg/ml), CaI(5μg/ml)	30	9.9*	68.0*	22.1
	Control	29	13.8	61.4	24.8

* p<0.05.

**Table II:** The effect of different concentration of selenium, calcium and calcium ionophore on GV oocytes *in* *vitro* (six replicated experiments).

**Interval (hours)**	**Treatments**	**No. Injected**	**Survival rate (%)**	**Fertilization rate (%)**
36				
	Se (5μg/ml), Ca (5 μg/ml), CaI (1μg/ml)	30	76.1*	71.6
	Se (10μg/ml), Ca (7 μg/ml), CaI(2μg/ml)	34	78.4*	70.9
	Se (15μg/ml), Ca (10μg/ml), CaI(5μg/ml)	31	81.6*	72.0
	Control	33	61.2	70.2
72				
	Se (5μg/ml), Ca (5 μg/ml), CaI (1 μg/ml)	31	78.4*	75.1
	Se(10μg/ml), Ca (7 μg/ml), CaI (2μg/ml)	35	79.8*	72.7
	Se (15μg/ml), Ca (10 μg/ml), CaI(5μg/ml)	34	84.4*	77.4
	Control	32	62.1	74.9
96				
	Se (5μg/ml), Ca (5 μg/ml), CaI (1 μg/ml)	34	80.7*	73.2
	Se(10μg/ml), Ca (7 μg/ml), CaI (2μg/ml)	30	83.1*	74.7
	Se (15μg/ml), Ca (10 μg/ml), CaI(5μg/ml)	30	82.2*	75.4
	Control	31	59.4	72.1

Se: Selenium , Ca: Calcium, CaI: Calcium Ionophore.

* p<0.05.

**Table III:** Embryo development of *in* *vitro* matured oocytes according to the interval between *in* *vitro* maturation and blastocyst formation.

**Treatments**	* **No of oocytes activated**	**% ** ** **oocytes** **cleaved**	**%** ** **oocytes developing** **beyond the morula stage**		**% ** ** **oocytes developing** **to the blastocyst**	
Se (5μg/ml), Ca (5 μg/ml), CaI (1 μg/ml)	32	62.3*		15.1*		7.1*
Se(10μg/ml), Ca (7 μg/ml), CaI (2 μg/ml)	34	66.1*		16.6*		9.5*
Se (15μg/ml), Ca (10 μg/ml), CaI (5 μg/ml)	34	76.2**		20.1**		11.1**
Control	30	50.1		10.3		5.4

Se: Selenium, Ca: Calcium, CaI: Calcium Ionophore.

*p<0.05 and **p<0.01.

* Oocytes were cultured for 24 h in chemically defined medium supplemented by Selenium, Calcium and Calcium Ionophore.

** Percentage based on the number of oocytes stimulated.

## Discussion

To our knowledge, this is the first study to investigate the effects of selenium, calcium and calcium ionophore supplementation in a chemically define medium to receive the highest rate of IVM. Selenium is an essential trace element which may have antioxidant activity in biological systems (21-23). Here, we used selenium in combination of calcium and calcium ionophore. Calcium elevation is a crucial event in triggering the complex machinery involved gamete maturation and fertilization (24, 25). 

Besides that, calcium ionophore, facilitates calcium current into the oocytes and helps the elevation of extracellular calcium concentration (26-28). The combination of these ions may be activating immature oocytes to receive the highest rate of maturation and fertilization. After counting the number of MI, MII and abnormal oocytes in different time lines, our data showed the highest percentage of MII oocytes (68%) after 24 hours treatment with 15µg/ml selenium, 10 µg/ml calcium and 5µg/ml calcium ionophore. 

Jeong *et al *(17) recommended the supplementation of the porcine IVM medium with 10 µg/ml insulin, 5.5 µg/ml transferrin and 5 µg/ml selenium. In addition, ITS plus L-ascorbic acid supplementation during the first 12 hours of in vitro maturation improves cytoplasm maturation (83.3%) in calf oocytes (18). 

Choi *et* *al* (29) showed the highest rate of oocytes activation (82%) in TCM-199, treated with 50 µmol calcium ionophore and cultured with cycloheximide for 24 hours. Furthermore, 61% of oocytes that had cultured with Calcium-EDTA for 48 hours formed a pro-nucleus (30). The percentage of MII oocytes was not significant in other time lines (8 and 16 hours), except the treatment with 15 µg/ml selenium, 10 µg/ml calcium and 5 µg/ml calcium ionophore after 16 hours. There was no difference between the percentages of abnormal oocytes in different combinations. It shows that these combinations do not cause to the abnormality in oocytes treated for in vitro maturation. The lowest percentage of abnormality was 22.1% among different treatments (Table I). 

In the next step, we assessed the normal fertilization and survival percentage following in vitro matured oocytes ICSI. There was no significant difference in percentage of normal fertilization. The highest percentage of fertilization was 77.4% in medium supplemented with 15 µg/ml selenium, 10 µg/ml calcium and 5 µg/ml calcium ionophore after 72 hours. As the same as fertilization rate, we observed the highest rate of survival (84.4%) in oocytes treated with 15 µg/ml selenium, 10 µg/ml calcium and 5 µg/ml calcium ionophore. The survival rate is significant in all combinations (Table II). Treatment with ITS plus porcine follicular fluid significantly improved the fertilization parameter of oocytes (17). 

At the final step, embryo development of in vitro matured oocytes evaluated. There was the significant difference between the percentages of oocytes that cleaved, developed beyond to the morula and blastocyst in all combinations, in compare to the chemically defined medium without treatment. The highest percentage of oocytes cleavage (76.2%) and developing beyond morula (20.1%) observed in medium supplemented with 15 µg/ml selenium, 10 µg/ml calcium and 5 µg/ml calcium ionophore. Furthermore, the highest percentage of blastocyst formation (11.1%) observed as the same group as later (Table III). When bovine oocytes cultured further in medium without Calcium-EDTA after 48 hours Calcium-EDTA supplementation, only 26% of the oocytes developed to the cleaved stage (30).

In conclusion, these results will allow future approaches on gamete in vitro maturation and the role of different ion concentrations on it. We need future studies to investigate the signaling pathways which involved in this process. 
